# Long-Term Persistence of Cell-Mediated and Humoral Responses to A(H1N1)pdm09 Influenza Virus Vaccines and the Role of the AS03 Adjuvant System in Adults during Two Randomized Controlled Trials

**DOI:** 10.1128/CVI.00553-16

**Published:** 2017-06-05

**Authors:** Robbert G. van der Most, Frédéric Clément, Julie Willekens, Walthère Dewé, Karl Walravens, David W. Vaughn, Geert Leroux-Roels

**Affiliations:** a GSK Vaccines, Rixensart, Belgium; b Center for Vaccinology, Ghent University and University Hospital, Ghent, Belgium; c GSK Vaccines, Rockville, Maryland, USA; Vanderbilt University Medical Center

**Keywords:** AS03 adjuvant, influenza vaccine, pandemic influenza

## Abstract

We investigated the role of AS03_A_ (here AS03), an α-tocopherol oil-in-water emulsion-based adjuvant system, on the long-term persistence of humoral and cell-mediated immune responses to A(H1N1)pdm09 influenza vaccines. In two studies, a total of 261 healthy adults (≤60 years old) were randomized to receive two doses of AS03-adjuvanted vaccine containing 3.75 μg of hemagglutinin (HA) or nonadjuvanted vaccine containing 15 μg of hemagglutinin (in study A) or 3.75 μg of hemagglutinin (in study B) 21 days apart. Hemagglutination inhibition (HI) antibody, memory B-cell, and CD4^+^/CD8^+^ T-cell responses were characterized up to 1 year following dose 1. We also assessed the effects of age and seasonal influenza vaccination history. AS03-adjuvanted (3.75 μg HA) vaccine and nonadjuvanted vaccine at 15 μg but not at 3.75 μg HA elicited HI antibody responses persisting at levels that continued to meet European licensure criteria through month 12. At month 12, the geometric mean titer for AS03-adjuvanted vaccine was similar to that for nonadjuvanted (15-μg) vaccine in study A (1:86 and 1:88, respectively) and higher than that for nonadjuvanted (3.75-μg) vaccine in study B (1:77 and 1:35, respectively). A(H1N1)pdm09-specific CD4^+^ T-cell and B-cell responses were stronger in AS03-adjuvanted groups and persisted only in these groups for 12 months at levels exceeding prevaccination frequencies. Advancing age and a seasonal vaccination history tended to reduce HI antibody and memory B-cell responses and, albeit less consistently, CD4^+^ T-cell responses. Thus, AS03 seemed to enhance the persistence of humoral and cell-mediated responses to A(H1N1)pdm09 vaccine, allowing for antigen sparing and mitigating potential negative effects of age and previous seasonal vaccination. (These studies have been registered at ClinicalTrials.gov under registration no. NCT00968539 and NCT00989287.)

## INTRODUCTION

Swine origin A(H1N1)pdm09 virus was the causative agent of the worldwide influenza pandemic in 2009-2010, which triggered the development of several pandemic A(H1N1)pdm09 influenza vaccines (1–3). An A(H1N1)pdm09 vaccine containing 3.75 μg of hemagglutinin (HA) adjuvanted with the α-tocopherol- and squalene-based oil-in-water emulsion adjuvant system AS03 (4) had a clinically acceptable safety profile in clinical studies and met both the European and U.S. regulatory guidance criteria for pandemic influenza vaccines in various populations (3, 5, 6). The A(H1N1)pdm09 virus continues to circulate as a seasonal influenza virus and has been included as the H1N1 strain in seasonal influenza vaccines since its appearance. Sustained immune responses elicited by A(H1N1)pdm09 vaccines should be boostable to putatively protective levels by either revaccination or administration of a seasonal vaccine containing the same viral antigens, particularly in seasons when A(H1N1)pdm09 is the predominant circulating strain (7).

Preexisting and vaccine-induced memory B and T cells define the quality and quantity of vaccine-induced humoral immune responses (8, 9). In previous trials, prior seasonal influenza vaccination and advancing age of the (adult) vaccinee were shown to be negative determinants of the humoral and/or cell-mediated immune (CMI) responses to A(H1N1)pdm09 vaccine (3, 5, 6, 10, 11). We previously reported the results of two studies evaluating A(H1N1)pdm09 vaccines in nonelderly adults (designated studies A and B [ClinicalTrials.gov registration no. NCT00968539 and NCT00989287]) (6). The studies examined the effects of AS03_A_ (here AS03) on the hemagglutination inhibition (HI) antibody responses and, in study A only, on CD4^+^ T-cell responses, up to 3 weeks after the second dose (day 42). The effects of a history of seasonal influenza vaccination on the postvaccination HI antibody responses, as well as vaccine safety, were also evaluated up to day 42. The AS03-adjuvanted vaccine assessed contained 3.75 μg of HA (in both studies), and the nonadjuvanted vaccines contained either 15 μg of HA (the standard seasonal dosage; in study A) or 3.75 μg of HA (in study B). AS03 was shown to enhance the vaccine-induced A(H1N1)pdm09-specific HI antibody and CD4^+^ T-cell responses up to day 42. We also showed that a single dose of any of the three vaccines sufficed to induce HI responses exceeding the European Medicines Agency Committee for Human Medicinal Products (CHMP) immunologic licensure criteria (12).

Supplementing the earlier data, the present report describes the persistence of the vaccine-induced A(H1N1)pdm09-specific HI antibody T-cell and memory B-cell responses at 6 months (the expected transmission period for influenza virus during one season) and 12 months following the first dose in the same populations. These responses were evaluated overall and stratified by the participants' previous receipt of seasonal influenza vaccination, their age, or both. We also describe the vaccine safety data through the end of the study.

(The work for both studies was presented in part at the Options for the Control of Influenza VII conference, Hong Kong SAR, China, 3 to 7 September 2010, and the Nationale Impfkonferenz, Stuttgart, Germany, 8 and 9 February 2011.)

## RESULTS

### Population demographics.

**FIG 1:**
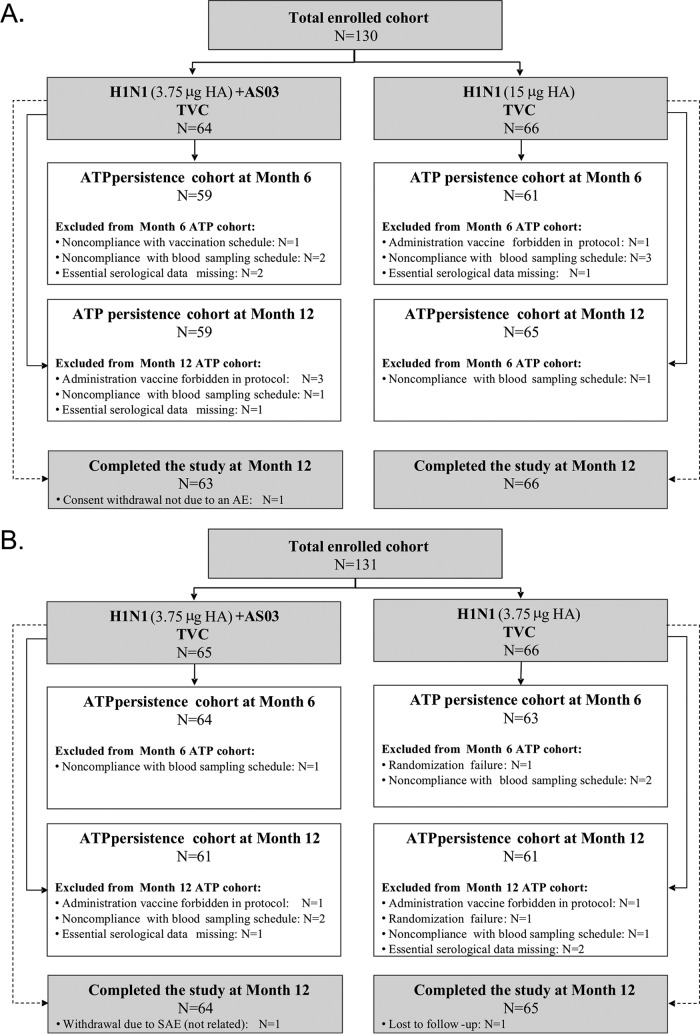
Participant flowcharts for studies A and B. H1N1, A(H1N1)pdm09 vaccine. N, number of participants.

The 130 and 131 subjects in studies A and B, respectively, were included in the total vaccinated cohort (TVC). Of these subjects, 10 and 6 in study A, and 4 and 9 in study B, were excluded from the according-to-protocol (ATP) cohorts at months 6 and 12, respectively, for the reasons specified in Fig. 1. In study A, all subjects completed the study except for one recipient of adjuvanted vaccine who withdrew consent (not because of an adverse event [AE]) prior to dose 2. In study B, all but two subjects completed the study (i.e., one recipient of adjuvanted vaccine withdrew because of a serious AE [SAE], as described below, and one recipient of nonadjuvanted vaccine was lost to follow-up).

The demographic profiles observed at month 12 were similar in studies A and B in terms of mean age (39.3 and 38.2 years, respectively), gender (∼60% females in both studies), and geographic ancestry (99.2% Caucasian heritage in both studies; Table 1). The profiles were also balanced between the vaccine groups, except that the number of subjects with a history of seasonal influenza vaccination was higher in study A than in study B (49.2 and 28.7%, respectively).

**TABLE 1:** Demographic profiles of the per-protocol cohorts for immunogenicity at month 12

Parameter	Study A			Study B		
	H1N1^*d*^/AS03	H1N1	Overall	H1N1/AS03	H1N1	Overall
No. of subjects	59	65	124	61	61	122
18–40 yr old	27	32	59	30	29	59
41–60 yr old	32	33	65	31	32	63
Mean age (yr) ± SD^*a*^						
18–40 yr old	27.2 ± 6.6	25.7 ± 6.6	26.4 ± 6.6	24.0 ± 4.8	26.3 ± 5.7	25.1 ± 5.3
41–60 yr old	51.4 ± 4.7	50.7 ± 5.9	51.0 ± 5.3	50.3 ± 5.0	50.8 ± 6.5	50.5 ± 5.7
18–60 yr old	40.3 ± 13.4	38.4 ± 14.1	39.3 ± 13.7	37.3 ± 14.1	39.1 ± 13.7	38.2 ± 13.9
Gender (% female)	61	60	60.5	67.2	52.5	59.8
Geographic ancestry (%)						
Asian	0	1.5	0.8	0	1.6	0.8
Caucasian	100	98.5	99.2	100	98.4	99.2
History of influenza vaccination; *n* (%)^*b*^						
18–40 yr old	9 (33.3)	5 (15.6)	14 (23.7)	NA^*c*^	NA	NA
41–60 yr old	26 (81.3)	21 (63.6)	47 (72.3)	NA	NA	NA
18–60 yr old	35 (59.3)	26 (40.0)	61 (49.2)	19 (31.1)	16 (26.2)	35 (28.7)

a Reported at the first dose.

b Reported as having received seasonal influenza vaccination at least once during the preceding 3 years.

c NA, data not available.

d H1N1, A(H1N1)pdm09 vaccine.

### Immunogenicity. (i) A(H1N1)pdm09-specific HI antibody responses.

At months 6 and 12, the HI antibody titers in both studies had decreased substantially from their peaks at day 42 but persisted at levels exceeding those at prevaccination (time zero; Table 2). At month 12, the geometric mean titers (GMTs) in the adjuvanted and nonadjuvanted groups were comparable in study A (86 [range, 64 to 114]) and 88 ([range, 65 to 119], respectively) but different in study B, where both vaccines contained the same antigen dose (77 [range, 58 to 102] and 35 [range, 24 to 52], respectively). Similar results were observed for the seroprotection rate (SPR), seroconversion rate (SCR), and geometric mean fold rise (GMFR). Consequently, the CHMP guidance criteria were all still met at month 6 in both studies and at month 12 by both groups in study A and by the adjuvanted group in study B. In the nonadjuvanted group in study B, however, the SPR and SCR (43 and 36%, respectively) no longer exceeded the respective criteria at month 12.

**TABLE 2:**
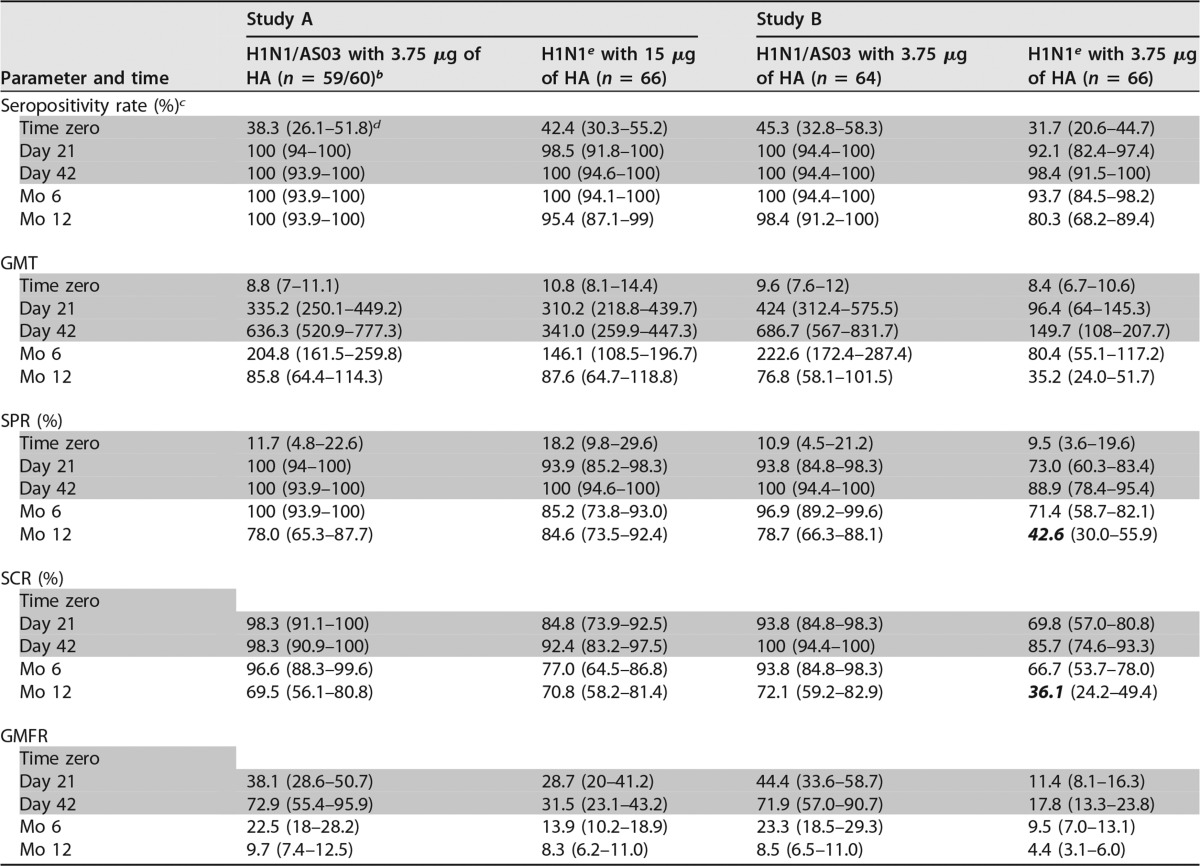
A(H1N1)pdm09-specific HI responses before and after vaccination up to month 12^*a*^

a Gray-shaded data were reported previously (6) and are shown here for completeness. The data are from the per-protocol cohorts for immunogenicity at day 42, month 6, and month 12. Bold italic values are those that do not meet the European Medicines Agency CHMP criteria for HI antibody responses in adults 18 to 60 years old, i.e., an SPR of >70%, an SCR of >40%, and a GMFR of >2.5.

^b^ *n* = 60 for time zero and day 21; *n* = 59 for all subsequent time points.

^c^ The seropositivity rate is the percentage of subjects with HI titers within the specified range.

^d^ Values in parentheses are 95% CIs.

^e^ H1N1, A(H1N1)pdm09 vaccine.

**FIG 2:**
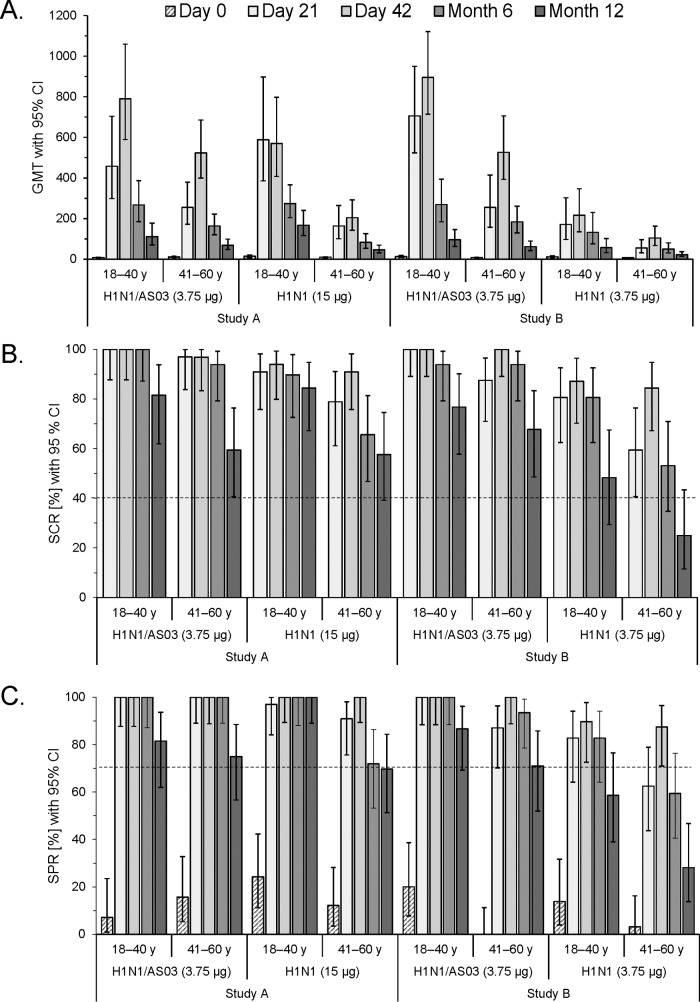
Age-stratified A(H1N1)pdm09-specific HI antibody responses. The GMTs (A), SCRs (B), and SPRs (C) with 95% CIs determined following vaccination with AS03-adjuvanted or nonadjuvanted A(H1N1)pdm09 (H1N1) vaccine containing 3.75 or 15 μg of HA are shown. Dotted lines represent the cutoffs of the CHMP criteria for HI antibody responses in adults 18 to 60 years of age with respect to the SCR (>40%) and SPR (>70%). The data for time zero (day 0) and days 21 and 42 supplement previously published data for the cohorts without age stratification (6). y, years.

The age-stratified HI data showed that the GMTs in both studies tended to persist at higher levels in the younger subjects (Fig. 2A). At months 6 and 12, all three CHMP criteria, including the GMFR (data not shown), were met in all of the subgroups in study A except in the middle-aged recipients of nonadjuvanted vaccine (where the SPR was not met at month 12; Fig. 2B and C). All criteria were also met in both subgroups receiving adjuvanted vaccine in study B. In contrast, the SCR and SPR criteria were no longer met by subsets of the nonadjuvanted group in study B on several occasions (i.e., by younger subjects at month 12 [SPR] and by middle-aged subjects at months 6 [SPR] and 12 [SPR and SCR]).

**FIG 3:**
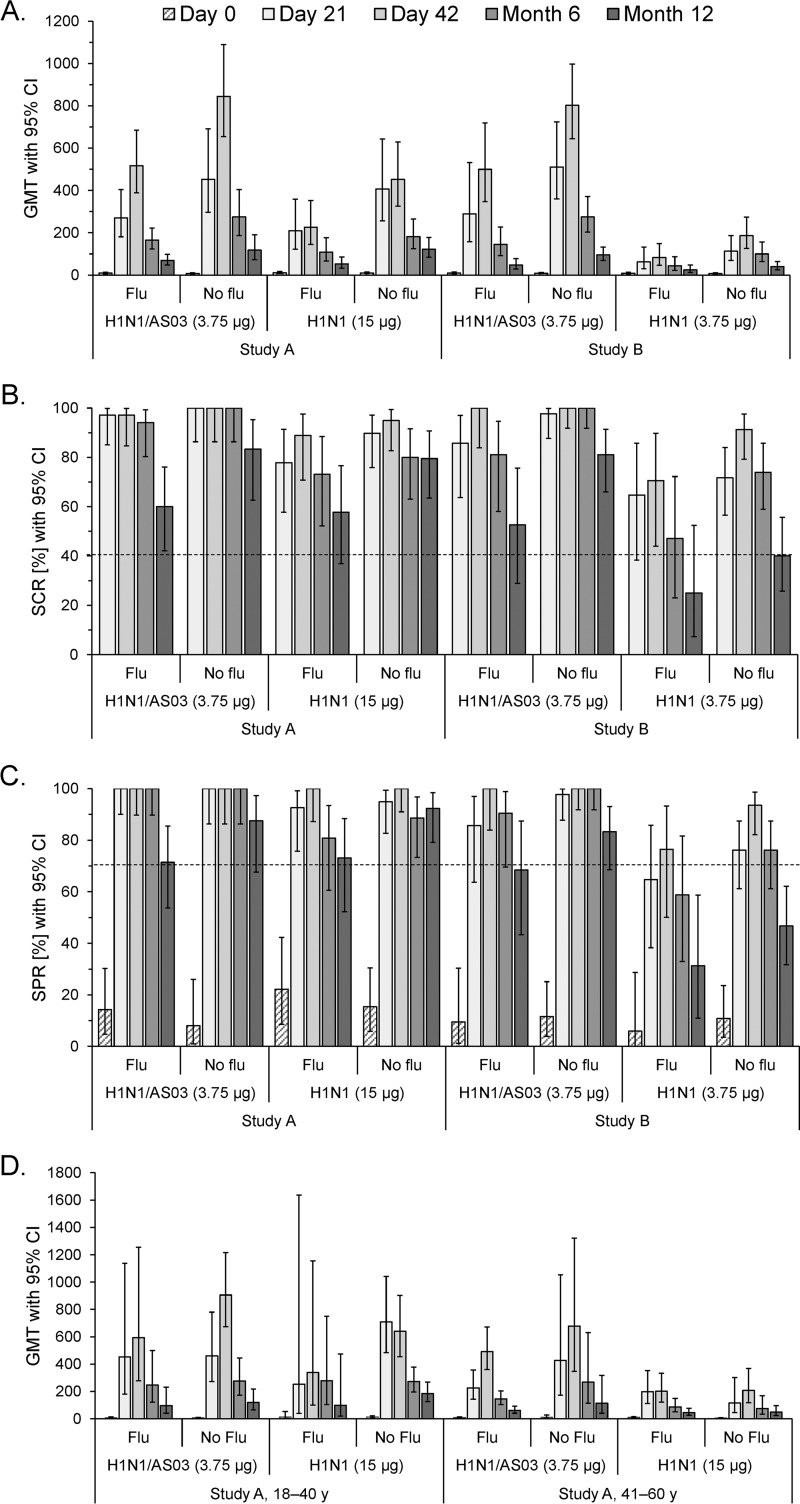
A(H1N1)pdm09-specific HI antibody responses stratified by the participants' history of seasonal influenza vaccination. The graphs shown represent the GMTs (A, D), SCRs (B), and SPRs (C) with 95% CIs determined following vaccination with the AS03-adjuvanted or nonadjuvanted A(H1N1)pdm09 (H1N1) vaccine. Data are stratified by the participants' history of seasonal influenza vaccination (Flu/No flu). The GMTs in study A are also stratified by age (D). Dotted lines represent the cutoffs of the CHMP criteria for the HI antibody responses in adults 18 to 60 years of age with respect to the SCR (>40%) and SPR (>70%). Data for time zero (day 0) and days 21 and 42 were reported previously (6) and are shown here for completeness. y, years.

Although the confidence intervals (CIs) overlapped, we observed a consistent trend for higher GMTs in the participants without a seasonal influenza vaccination history than in those with such a history in each vaccine group and in both studies (Fig. 3A). At months 6 and 12, all of the CHMP criteria, including the GMFR (data not shown), were exceeded by all of the subgroups in study A and in study B by recipients of adjuvanted vaccine without prior seasonal vaccination (Fig. 3B and C). In the remaining three subgroups in study B, one or more CHMP criteria were not met at months 6 and/or 12. Further stratification of the GMTs of study A by age revealed that at month 12, the negative effect of prior seasonal vaccination tended to be stronger in the nonadjuvanted groups among younger subjects (GMT [CI] with and without prior vaccination, 99 [21 to 474] and 184 [127 to 268] compared with 97 [40 to 233] and 120 [66 to 218] in the adjuvanted groups, respectively; Fig. 3D). Conversely, among the middle-aged subjects, this effect tended to be stronger in the adjuvanted groups (GMT [CI] at month 12 with and without prior vaccination, 61 [41 to 92] and 113 [41 to 317], compared with 46 [27 to 77] and 49 [25 to 97] in the nonadjuvanted groups, respectively). However, some of the sample sizes in these subgroups were small at month 12 (i.e., *n* = 5 or 6).

### (ii) T-cell responses.

A(H1N1)pdm09-specific CD4^+^ and CD8^+^ T cells expressing two or more immune markers (among CD40L, interleukin-2 [IL-2], tumor necrosis factor alpha [TNF-α], and gamma interferon [IFN-γ]) were evaluated after stimulation with either vaccine-homologous H1N1 split antigen or a pool of peptides spanning the A(H1N1)pdm09 HA. Responses of CD4^+^ T cells expressing two or more immune markers up to day 42, as evaluated in the nonstratified population of study A, were presented previously (6). In the present study, the CD4^+^ T cells elicited were shown to express primarily IL-2 but also IFN-γ and TNF-α (see Fig. S1 in the supplemental material; shown for study A).

**FIG 4:**
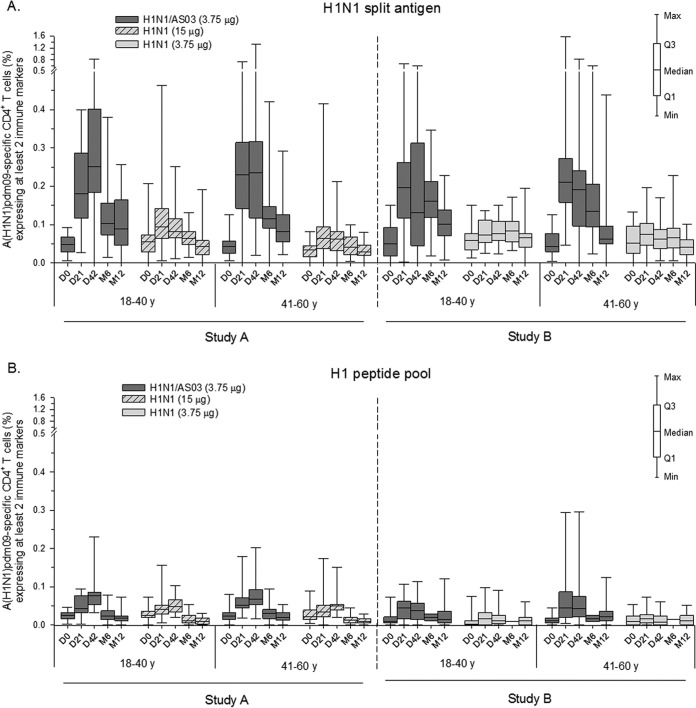
Age-stratified A(H1N1)pdm09-specific CD4^+^ T-cell responses. The graphs shown represent antigen-specific CD4^+^ T-cell responses induced by the AS03-adjuvanted or nonadjuvanted A(H1N1)pdm09 (H1N1) vaccine measured after stimulation with H1N1 split antigen (A) or the H1 pool of peptides (B). Data are reported as percentages of A(H1N1)pdm09-specific CD4^+^ T cells expressing at least two immune markers (IFN-γ, IL-2, TNF-α, or CD40L). Data for time zero (D0), day 21 (D21), and day 42 (D42) in study A supplement previously published data for the cohorts without age stratification (6). y, years. M, month. Q1 and Q3, first and third quartiles. Min and Max, minimum and maximum values.

Using split-antigen stimulation, low frequencies of preexisting A(H1N1)pdm09-specific CD4^+^ T cells were observed in all of the age-stratified subgroups in both studies (Fig. 4A). The data were suggestive of an adjuvant effect, regardless of the HA content of the nonadjuvanted vaccine used in comparison. Indeed, responses to the first or second dose of adjuvanted vaccine contracted steadily from their peaks (medians, ≤0.25%) through month 12 but remained above prevaccination levels. In contrast, the responses to both nonadjuvanted vaccines were either absent or low (medians, ≤0.10%) and did not persist in any of the subgroups at month 12. No obvious effect of age on pre- and postvaccination responses was observed in either vaccine group. Using H1 peptide pool stimulation, frequencies were overall low (medians, ≤0.08%) and exhibited trends similar to those observed when using the split-antigen stimulation (Fig. 4B).

**FIG 5:**
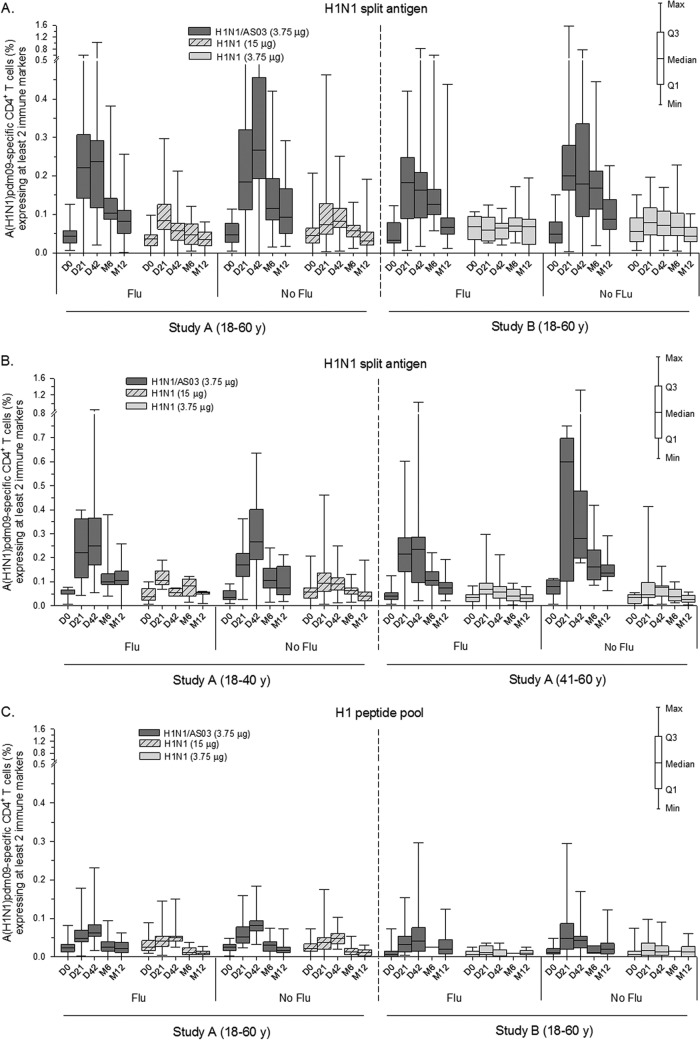
A(H1N1)pdm09-specific CD4^+^ T-cell responses stratified by the participants' history of seasonal influenza vaccination and age. The graphs shown represent antigen-specific CD4^+^ T-cell responses induced by the AS03-adjuvanted or nonadjuvanted A(H1N1)pdm09 (H1N1) vaccine measured after stimulation with H1N1 split antigen (A and B) or the H1 pool of peptides (C). Data are stratified by the participants' history of seasonal influenza vaccination (Flu/No flu; panels A to C) and by age (panel B; study A only) and reported as percentages of A(H1N1)pdm09-specific CD4^+^ T cells expressing at least two immune markers (IFN-γ, IL-2, TNF-α, or CD40L). Data for time zero (D0), day 21 (D21), and day 42 (D42) in study A supplement previously published data for the cohorts without stratification for seasonal influenza vaccination history (6). y, years. M, month. Q1 and Q3, first and third quartiles. Min and Max, minimum and maximum values.

In both studies, CD4^+^ T-cell responses to the adjuvanted vaccine were comparable between the seasonal vaccination subgroups both before and after vaccination (Fig. 5A; shown for the split-antigen stimulation). Interestingly, when the data of study A were further stratified by age, there was, among the middle-aged recipients of adjuvanted vaccine, a trend for stronger responses in participants without prior seasonal vaccination versus participants with prior seasonal vaccination (i.e., median [interquartile range; IQR] at day 21, 0.60% [0.10 to 0.70] and 0.22% [0.14 to 0.28]), which was still observed at month 12 (i.e., 0.14% [0.12 to 0.17] and 0.07% [0.05 to 0.10], respectively; Fig. 5B). Similarly, the responses in this subgroup also tended to be stronger than those of the younger recipients of adjuvanted vaccine, regardless of the seasonal vaccination history of the latter participants. Yet, for both observations, the small sample size of the subgroup of middle-aged recipients of adjuvanted vaccine without a seasonal vaccination history (*n* = 6) precludes any definitive conclusions. No effect of previous seasonal vaccination was observed following stimulation with the peptide pools (Fig. 5C).

**FIG 6:**
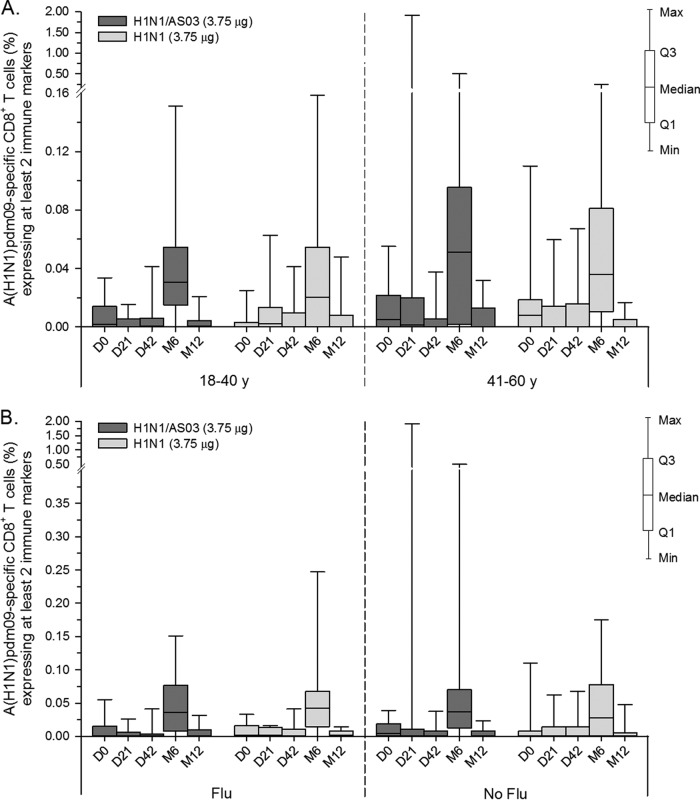
A(H1N1)pdm09-specific CD8^+^ T-cell responses in study B. The graphs shown represent antigen-specific CD8^+^ T-cell responses induced by the AS03-adjuvanted or nonadjuvanted A(H1N1)pdm09 (H1N1) vaccine measured after stimulation with H1N1 split antigen. Data are stratified either by age (A) or by the participants' history of seasonal influenza vaccination (Flu/No flu; B) and reported as percentages of A(H1N1)pdm09-specific CD8^+^ T cells expressing at least two immune markers (IFN-γ, IL-2, TNF-α, or CD40L). y, years. M, month. Q1 and Q3, first and third quartiles. Min and Max, minimum and maximum values.

Regardless of the stimulation method used, no apparent vaccine-elicited cytokine-positive CD8^+^ T-cell responses were detected in either study. However, a weak response (medians, ≤0.05%) was observed in a minority of subjects in both vaccine groups in study B at month 6 when using split-antigen stimulation (Fig. 6A and B; it is noted that the sample sizes for the peptide pool stimulation were small at this time point, i.e., *n* = 1 or 2/subgroup).

### (iii) Memory B cells (assessed in study B only).

**FIG 7:**
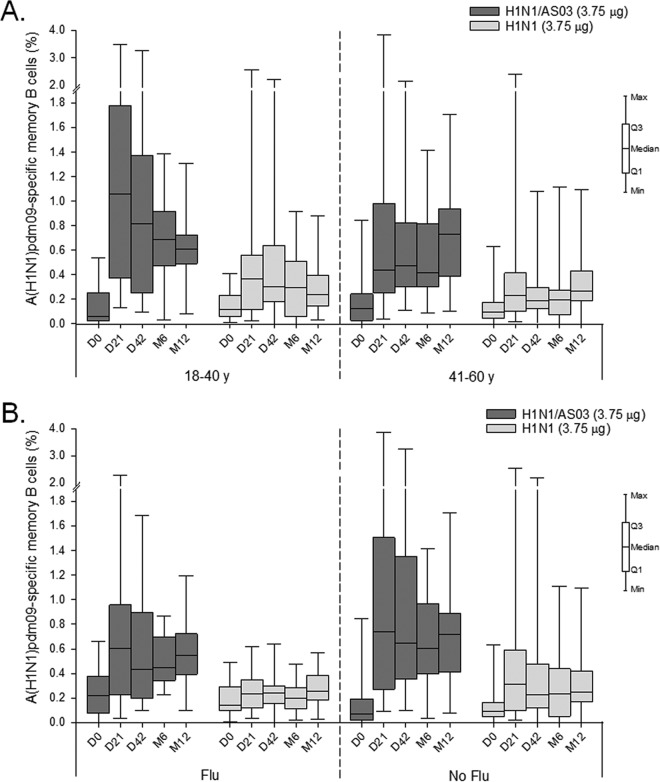
A(H1N1)pdm09-specific memory B-cell responses in study B. The graphs shown represent the frequencies of antigen-specific memory B cells in the total number of IgG-producing memory B cells induced by the AS03-adjuvanted or nonadjuvanted A(H1N1)pdm09 (H1N1) vaccine. The data are stratified either by age (A) or by the participants' history of seasonal influenza vaccination (Flu/No flu; B), y, years. M, month. Q1 and Q3, first and third quartiles. Min and Max, minimum and maximum values.

The data revealed an adjuvant effect in both age groups (Fig. 7A). Preexisting memory B-cell responses were similar in the two age groups. The postvaccination responses in the adjuvanted groups tended to be initially stronger in the younger than in the middle-aged subjects (median [IQR] at day 21, 1.06% [0.37 to 1.78%] and 0.44% [0.25 to 0.98%], respectively) but comparable between the two age groups at months 6 and 12. No age effect was observed in the nonadjuvanted groups. Preexisting responses were also similar between the seasonal vaccination subgroups (Fig. 7B). In the adjuvanted groups, there was a slight tendency for stronger postvaccination responses in subjects without previous seasonal vaccination, while in the nonadjuvanted groups, the responses were comparable between both seasonal vaccination subgroups.

Similar results were obtained in TVC-based analyses of both studies (data not shown), which were performed because the percentage of subjects excluded from the ATP cohort in the adjuvanted group exceeded 5%.

### Safety.

The observed incidences of unsolicited AEs (overall and grade 3) up to day 84 were comparable in the adjuvanted and nonadjuvanted vaccine groups (i.e., unsolicited AEs occurred after 41.7 and 38.6% of the doses in study A and 45.7 and 38.5% of the doses in study B and grade 3 unsolicited AEs occurred after 5.5 and 7.6% of the doses in study A and 6.2 and 6.9% of the doses in study B). Vaccination-related unsolicited AEs were more frequent in the adjuvanted groups than in the nonadjuvanted groups (after 11.0 and 5.3% of the doses in study A and 13.2 and 7.7% of the doses in study B, respectively). All reported vaccination-related grade 3 events occurred in the 42-day follow-up period of study A (6).

In study A, three subjects reported one SAE each up to month 12 (see Table S1). One of these SAEs was reported by a recipient of nonadjuvanted vaccine in the 42-day follow-up period (6). No subject withdrew because of an SAE. In study B, four subjects in each vaccine group reported at least one SAE each in the follow-up period up to month 12. One male recipient of adjuvanted vaccine, who was 59 or 60 years of age at the onset of the symptoms, developed an open wound and a renal disorder combined with sepsis at 315 and 341 days after the second dose, respectively, and did not return for the month 12 visit. All SAEs resolved without sequelae or were resolving at the time of reporting (February 2011), with the exception of a balance disorder reported by a recipient of adjuvanted vaccine in study A, which was unresolved in February 2011. None of the SAEs were fatal or considered by the investigator as related to vaccination.

In study A, one recipient of adjuvanted vaccine reported an AE of specific interest (AESI), pharyngeal edema, twice (at days 0 and 20 after dose 1). Each event lasted 1 day. One recipient of nonadjuvanted vaccine reported a potential immune-mediated disease (pIMD), uveitis, at 48 days after dose 2, that lasted 9 days and was not considered autoimmune in origin. None of these events were considered by the investigator to be related to vaccination. Neither AESIs nor pIMDs were reported in study B.

## DISCUSSION

Previously, we presented the HI antibody and CD4^+^ T-cell responses elicited by two doses of AS03-adjuvanted or nonadjuvanted A(H1N1)pdm09 influenza vaccine up to day 42 in two populations of adults ≤60 years of age (6). These data were completed by the present results describing the long-term persistence of the HI antibody, CD4^+^ and CD8^+^ T-cell, and memory B-cell responses up to month 12 in the same populations. Given the increasing interest in understanding the effect of the AS03 adjuvant system on long-term immune memory, we generated the present data set to further this understanding. Responses were evaluated overall and stratified by previous receipt of seasonal influenza vaccines and/or age. While no intergroup statistical comparisons were performed, these data suggest that (i) two doses of adjuvanted vaccine (3.75 µg HA) or nonadjuvanted vaccine at 15 μg of HA but not at 3.75 μg of HA elicited HI antibody responses persisting at levels meeting CHMP criteria through month 12; (ii) advancing age and a history of seasonal influenza vaccination tended to reduce the HI antibody and memory B-cell responses, while such effects were less consistently observed for the CD4^+^ T-cell responses; and (iii) the use of AS03 may have enhanced the persistence of vaccine responses of both arms of the immune system, thus also mitigating (at least partially) any potential negative effects of age and previous seasonal vaccination.

While contrasting reports exist (13), the reducing effect of previous seasonal vaccination on HI responses to AS03-adjuvanted or nonadjuvanted A(H1N1)pdm09 vaccines has been well described in different populations (2, 3, 5, 11, 14–22), and the present data add to the existing literature. Our results also align with the data for these studies' populations up to day 42 (6). The observation that the persisting memory B-cell responses to the AS03-adjuvanted vaccine may have been similarly affected by the immune interference between seasonal and pandemic influenza vaccines contrasts with an earlier study with this vaccine (21) but aligns with data for a similar AS03-adjuvanted A(H1N1)pdm09 vaccine (22). Several authors have attributed these diminishing effects to the “original antigenic sin” phenomenon, implying that the memory B-cell pool specific to the seasonal influenza epitopes, as induced by seasonal vaccination, has become less proficient in adapting to the A(H1N1)09pdm antigens in the subsequent pandemic influenza vaccine (9, 23). It has been suggested that the effect can be counteracted by oil-in-water adjuvants such as AS03, possibly by stimulating CD4^+^ T-cell responses, including responses of T follicular helper cells (9, 24, 25).

Age as a negative determinant of the HI response to A(H1N1)pdm09 vaccines is well described in the elderly (3, 10, 13, 15) and, albeit less extensively, in nonelderly adults (13, 15, 26–28). This, in conjunction with the age-related decline in the HI and memory B-cell responses observed here, indicates that vaccine responses are heterogeneous between these age segments in nonelderly adults. This observation is consistent with recently published systems biology data (29). Interestingly, we observed that the compensation by AS03 of the negative effects on the memory B-cell responses was more apparent for the effects of seasonal vaccination than for age-related effects, which could suggest that the latter effect was relatively stronger. The enhancement of long-term persisting HI or memory B-cell responses to the A(H1N1)pdm09 antigen by AS03, which likely resulted from the adjuvant effect on the CD4^+^ T-cell response (9), supplements comparable results from a variety of studies performed in adults (10, 15, 21, 22, 30), and this effect has also been shown for plasmablast responses (22). Besides their effect on the quantitative age-related changes in the immune response, oil-in-water-based adjuvants are also known to improve the functional age-related changes such as a reduced binding affinity of antibodies (31, 32).

No obvious effect on the persistence of CD4^+^ T-cell responses was seen for age and prior vaccination history when these determinants were evaluated individually. This is consistent with previous data for this vaccine (21) but possibly contradicts other data for a similar vaccine (22). However, after stratifying the data by both determinants simultaneously, we observed that for the recipients of adjuvanted vaccine without prior seasonal vaccination, the preexisting, peak, and longer-term persisting CD4^+^ T-cell responses tended to be stronger in the middle-aged subjects than in the younger subjects. For adults, it has been reported that the preexisting immune responses to influenza vaccination, more than aging, determine their vaccine-induced responses (reviewed in reference 33). We hypothesize that the middle-aged subjects in our study were more likely than the younger subjects to possess preexisting cross-reactive memory responses to A(H1N1)pdm09 antigens from serial A(H1N1) influenza infections, which may have resulted in stronger postvaccination responses to A(H1N1)pdm09 vaccine antigens, an effect that was previously reported for antibody responses (34). However, such a difference between the age groups was not observed in the A(H1N1)pdm09-specific antibody titers before or after vaccination (note that baseline anti-H1N1 antibody responses were not measured), and no correlation between baseline CD4^+^ T-cell frequencies and day 21 HI titers was previously detected (6). Moreover, for the IFN-γ^+^ CD4^+^ T-cell responses to a trivalent inactivated influenza vaccine in adults, the fold increases from the baseline after vaccination were previously shown to correlate inversely with the baseline responses (35), further confounding this observation.

After the H1N1 split-antigen stimulation, CD8^+^ T-cell responses were observed in both vaccine and age groups at month 6 only (which occurred in the month of April). By study protocol, subjects with a clinical history suggestive of an influenza virus infection within 6 months before the study start were excluded. Despite these stringent criteria, we cannot dismiss the possibility that a few subjects may have had an intercurrent infection with a seasonal strain other than H1N1 in the preceding winter season that went unnoticed or caused only minor cold-like symptoms. Such infections may have evoked cross-reactive responses between conserved proteins of the seasonal and A(H1N1)pdm09 vaccine strains, e.g., nucleoprotein or matrix proteins (34). Any cross-reactive responses would then be detected after split-antigen stimulation, which included the whole virus, rather than with peptide stimulation, which only included surface H1 antigens.

A possible study limitation was that sample sizes for T-cell evaluations, particularly at month 6, were too limited to draw definitive conclusions between the subgroups. This was a result of the relatively large blood sample volumes required for the elaborate CMI response assessments. In addition, it is noted that given the potential presence of sex-based differences in the immune responses, as reported previously (36–38), the present conclusions may not apply equally to both genders.

No safety concerns were observed in the present studies. In retrospective observational studies, an increased incidence of narcolepsy was observed in children and adolescents after administration of the same adjuvanted A(H1N1)pdm09 vaccine as the one that was evaluated here (39–42). However, the present studies were not powered to detect rare events such as narcolepsy and no cases were observed.

In conclusion, two doses of the AS03-adjuvanted A(H1N1)pdm09 vaccine (3.75 µg HA) or two doses of nonadjuvanted A(H1N1)pdm09 vaccine at 15 µg HA, but not of the nonadjuvanted vaccine at 3.75 µg HA, elicited HI immune responses meeting the CHMP regulatory guidance criteria up to 1 year after vaccination, as well as long-term persisting memory B-cell and CD4^+^ T-cell responses, in adults 18 to 60 years of age. While age and recent seasonal influenza vaccination tended to reduce HI antibody, memory B-cell, and, to a lesser extent, CD4^+^ T-cell responses, these effects appeared to be partially or completely compensated for by the use of AS03.

## MATERIALS AND METHODS

### Study design.

Studies A and B were randomized (1:1), observer-blind, single-center, phase III studies conducted from September 2009 to December 2010 in Ghent, Belgium, in accordance with the Helsinki Declaration and Good Clinical Practices (ClinicalTrials.gov registration no. NCT00968539 and NCT00989287, respectively) (6).

In each study, two parallel groups of adults 18 to 60 years of age received two doses of either AS03-adjuvanted or nonadjuvanted A(H1N1)pdm09 vaccine according to a day 0 and week 3 schedule. Subjects with a clinical history suggestive of an influenza virus infection within 6 months preceding the study start were excluded. For the present evaluations, the subjects were stratified (1:1) by age into young (18 to 40 years old) and middle-aged (41 to 60 years old) categories. Overall (and in each age category in study A), the subjects were also grouped by having a history of seasonal influenza vaccination within the three seasons prior to vaccination (the 2007-2008, 2008-2009, and 2009-2010 seasons).

Blood samples for immunogenicity assessments were collected prior to each dose (days 0 and 21), 3 weeks after dose 2 (day 42), and 6 and 12 months following dose 1 (months 6 and 12).

### Study objectives.

As reported previously (6), the primary objective of study A was to demonstrate that two doses of AS03-adjuvanted A(H1N1)pdm09 vaccine result in a vaccine HA-homologous HI antibody response meeting the CHMP guidance criteria (12) at day 42, and that of study B was to assess the HI antibody response to A(H1N1)pdm09 vaccine (3.75 μg of HA) with and without AS03, in terms of the CHMP criteria at day 21. The secondary objectives of both studies were to describe the HI antibody responses up to month 12 in the study population and to demonstrate vaccine safety.

The primary objectives of the present report were to describe the persistence of the immune response to adjuvanted and nonadjuvanted vaccines at months 6 and 12 in terms of HI antibody responses and, as part of the exploratory study objectives, CD4^+^/CD8^+^ T-cell responses (both studies) and memory B-cell responses (study B) in the study populations (either not stratified or stratified by age stratum and/or by history of prior seasonal influenza vaccination).

### Study vaccines.

The AS03-adjuvanted A(H1N1)pdm09 vaccine (Pandemrix; GSK Vaccines, Dresden, Germany) was a monovalent split-virion, inactivated influenza vaccine containing 3.75 μg of HA of A/California/7/2009 (H1N1) NYMC X-179A (43). The nonadjuvanted A(H1N1)pdm09 vaccines contained 15 μg of HA in study A and 3.75 μg of HA in study B. AS03_A_ (elsewhere in this report referred to as AS03) is an α-tocopherol- and squalene-based oil-in-water emulsion adjuvant system containing 11.86 mg of tocopherol per dose (4).

### Immunogenicity evaluations. (i) Humoral immune responses.

Serum samples were tested with a validated HI microtiter assay using chicken erythrocytes (44) and with the A(H1N1)pdm09 vaccine strain used as the antigen. Results are expressed as reciprocal titers.

### (ii) T-cell responses.

CD4^+^ and CD8^+^ T-cell frequencies were evaluated by intracellular cytokine staining and flow cytometry assays as described previously (6). *In vitro* stimulation of peripheral blood mononuclear cells was performed with either A/California/7/2009 H1N1 split antigen or A/California/7/2009 H1 HA peptide pools. Results are expressed as background (medium)-subtracted frequencies of antigen-specific CD4^+^/CD8^+^ T cells expressing two or more immune markers (CD40L, IFN-γ, IL-2, or TNF-α) in the total number of CD4^+^ or CD8^+^ T cells.

### (iii) Memory B-cell responses.

Memory B-cell frequencies were enumerated in study B with a memory B-cell enzyme-linked immunosorbent spot assay as described previously (45, 46). Results are expressed as percentages of A(H1N1)pdm09-specific memory B cells in the total number of immunoglobulin G-producing memory B cells.

### Safety evaluation.

Safety was evaluated for the TVC. Safety and reactogenicity data from the vaccination phase and the 21-day follow-up were reported previously (6). The occurrence of/relationship to vaccination of all unsolicited AEs over the 84-day follow-up period, as well as SAEs, AESIs, pIMDs, and withdrawals due to AEs through month 12, are described in the present report.

### Statistical methods.

The immunogenicity data were reported descriptively, overall for all subjects, by age group, and/or by history of seasonal influenza vaccination. Computations were performed with SAS 9.1.3.

Evaluation of HI antibody responses of the ATP cohorts for immunogenicity at day 42 (described in reference 6), month 6, or month 12 was performed. The humoral immune response was first characterized by the standard statistical parameters used by the CHMP for evaluation of influenza vaccines and then assessed by using the guidance targets for pandemic influenza vaccines in adults (point estimates of an SCR of >40%, an SPR of >70%, and a GMFR of >2.5) (12). By definition, the SCR is the percentage of subjects with a prevaccination titer of <1:10 and a postvaccination titer of ≥1:40 or a prevaccination titer of >1:10 and a ≥4-fold higher postvaccination titer; the SPR is the percentage of subjects with a serum HI titer of ≥1:40, and the GMFR is the geometric mean of the within-subject ratios of postvaccination reciprocal titer to the prevaccination reciprocal titer. The HI antibody response was further assessed by using geometric mean titers (GMTs) and seropositivity rates (percentages of subjects with titers of ≥1:10).

At each blood sampling time point, the CMI responses [in terms of frequencies of A(H1N1)pdm09-specific CD4^+^ or CD8^+^ T lymphocytes and A(H1N1)pdm09-specific memory B lymphocytes] were evaluated on the ATP cohort for immunogenicity at month 12.

Safety data were also reported descriptively. Incidences of the number of doses followed by ≥1 unsolicited AE or grade 3 unsolicited AE were tabulated with 95% CIs.

## Supplementary Material

Supplemental material
