# Supplementary material for: Long-Term Persistence of Cell-Mediated and Humoral Responses to A(H1N1)pdm09 Influenza Virus Vaccines and the Role of the AS03 Adjuvant System in Adults during Two Randomized Controlled Trials
Source: Clin Vaccine Immunol. 2017 Jun 5;24(6):e00553-16. doi: 10.1128/CVI.00553-16 (PMC5461372; doi:10.1128/CVI.00553-16)
Supplement: Supplemental material [file supp_24_6_e00553-16__index.html]

Supplemental material 

# Long-Term Persistence of Cell-Mediated and Humoral Responses to A(H1N1)pdm09 Influenza Virus Vaccines and the Role of the AS03 Adjuvant System in Adults during Two Randomized Controlled Trials

## Supplemental material

- Supplemental file 1 -

  Fig. S1. Immune marker expression of A(H1N1)pdm09-specific CD4+ T-cell responses (study A).

  PDF, 108K
- Supplemental file 2 -

  Table S1. Serious adverse events reported during the follow-up period through month 12.

  PDF, 169K
