# Supplementary material for: Long-Term Persistence of Cell-Mediated and Humoral Responses to A(H1N1)pdm09 Influenza Virus Vaccines and the Role of the AS03 Adjuvant System in Adults during Two Randomized Controlled Trials
Source: Clin Vaccine Immunol. 2017 Jun 5;24(6):e00553-16. doi: 10.1128/CVI.00553-16 (PMC5461372; doi:10.1128/CVI.00553-16)
Supplement: Supplemental material [file CVI.00553-16_zcd999095487s1.pdf]

Figure S1: Immune-marker expression of A(H1N1)pdm09-specific CD4$^+$ T-cell responses (Study A). Adults 18-60 years of age received AS03-adjuvanted A(H1N1)pdm09 ('H1N1') vaccine containing 3.75 µg hemagglutinin (HA), or non-adjuvanted H1N1 vaccine containing 15 µg HA, at Day (D) 0 and D21. Data for the per-protocol cohort at Month (M) 12 are reported as percentages of A(H1N1)pdm09-specific CD4$^+$ T cells expressing at least the indicated immune marker and another, among interferon-gamma (IFN-γ), interleukin-2 (IL-2), tumor necrosis factor-alpha (TNF-α), and CD40L per million CD4$^+$ T cells, with medians, first and third quartiles and the minimum/maximum values measured, after stimulation with H1N1 split antigen. Data for D0 and D42 supplements previously published data for the cohort evaluated in terms of CD4$^+$ T cells expressing at least 2 immune markers (1).

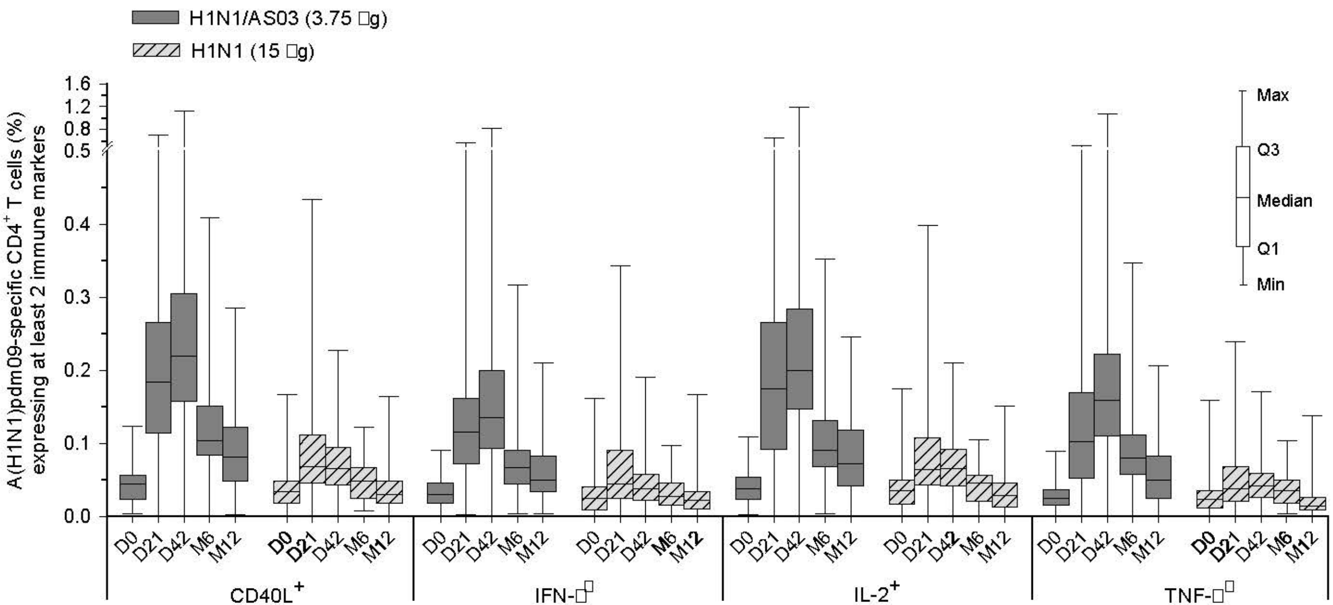

H1N1/AS03 (3.75 g)
H1N1 (15 g)
A(H1N1)pdm09-specific CD4+ T cells (%)
expressing at least 2 immune markers
1.6
1.2
0.8
0.5
0.4
0.3
0.2
0.1
0.0
Max
Q3
Median
Q1
Min
D0 D21 D42 M6 M12 D0 D21 D42 M6 M12
CD40L+
IFN-
IL-2+
TNF-

References

1. **Roman F, Clément F, Dewé W, Walravens K, Maes C, Willekens J, De Boever F, Hanon E, Leroux-Roels G**. 2011. Effect on cellular and humoral immune responses of the AS03 Adjuvant System in an A/H1N1/2009 influenza virus vaccine administered to adults during two randomized controlled trials. Clin Vaccine Immunol **18**:835-843.
