# Supplementary material for: Long-Term Persistence of Cell-Mediated and Humoral Responses to A(H1N1)pdm09 Influenza Virus Vaccines and the Role of the AS03 Adjuvant System in Adults during Two Randomized Controlled Trials
Source: Clin Vaccine Immunol. 2017 Jun 5;24(6):e00553-16. doi: 10.1128/CVI.00553-16 (PMC5461372; doi:10.1128/CVI.00553-16)
Supplement: Supplemental material [file CVI.00553-16_zcd999095487s2.pdf]

Supplementary Table 1. Serious adverse events (SAEs) reported during the follow-up period through Month 12.

Study	Treatment	Age at onset (years)	Gen-der	Description[1]	Dose	Day of onset[2]	Duration (days)	Outcome
A	H1N1/AS03 (3.75 µg HA)	27	F	Ectopic pregnancy	2	326	31	Resolved
		57	M	Spinal osteoarthritis	2	106	288	Resolved
	H1N1 (15 µg HA)	41	M	Migraine[3]	1	14	1	Resolved
B	H1N1/AS03 (3.75 µg HA)	59	F	Balance disorder	2	293	-	Not resolved[4]
		21	F	Spontaneous abortion	2	152	2	Resolved
		60	F	Breast cancer	2	85	130	Resolved
		59	M	Open wound*	2	315	72	Resolved
		60	M	Renal disorder*	2	341	46	Resolved
		60	M	Sepsis*	2	341	46	Resolved
	H1N1 (3.75 µg HA)	23	F	Asthma	2	165	12	Resolved
		61	M	Benign prostatic hyperplasia[#]	2	297	20	Resolved
		61	M	Calculus ureteric[#]	2	297	20	Resolved
		61	M	Nephrolithiasis[#]	2	297	20	Resolved
		21	M	Radius fracture	2	95	105	Resolved
		47	F	Ligament rupture	2	127	-	Resolving[4]

H1N1, A(H1N1)pdm09 vaccine. HA, hemagglutinin. M, male. F, female. [1]SAEs marked with the same symbol (* or [#]) were reported by the same subject. [2]Number of days since last study vaccine dose. [3]Reported previously in (1). [4]Status in February 2011 (reporting date).

Reference

1. **Roman F, Clément F, Dewé W, Walravens K, Maes C, Willekens J, De Boever F, Hanon E, Leroux-Roels G**. 2011. Effect on cellular and humoral immune responses of the AS03 Adjuvant System in an A/H1N1/2009 influenza virus vaccine administered to adults during two randomized controlled trials. Clin Vaccine Immunol 18:835-843.
